# Integrative approaches in Alzheimer’s disease: evaluating the potential of traditional, complementary, and integrative medicine (TCIM)

**DOI:** 10.3389/fphar.2025.1561702

**Published:** 2025-06-30

**Authors:** R. Sneha Sri, T. Pavithra, T. Vinciya, V. Santhosh Kumar, N. Harikrishnan, Rukaiah Fatma Begum, S. Ankul Singh

**Affiliations:** ^1^ Department of Pharmacology, Dr. M.G.R. Educational and Research Institute, Chennai, Tamil Nadu, India; ^2^ Department of Pharmaceutical Analysis, Dr. M.G.R. Educational and Research Institute, Chennai, Tamil Nadu, India; ^3^ Institute of Pharmaceutical Research, GLA University, Mathura, Uttar Pradesh, India

**Keywords:** Alzheimer’s disease (AD), traditional medicine, complementary and integrative medicine (TCIM), herbal therapies, neuroinflammation, cognitive decline

## Abstract

This review explores the potential of Traditional, Complementary, and Integrative Medicine (TCIM) as an adjunct to conventional therapies for Alzheimer’s Disease (AD). Unlike pharmaceutical treatments that primarily offer symptomatic relief, TCIM encompasses holistic approaches that target multiple pathophysiological pathways involved in AD, including tau pathology, oxidative stress, mitochondrial dysfunction, and neuroinflammation. Herbal therapies such as *Withania somnifera*, *Ginkgo biloba*, and *Curcuma longa* have shown promising neuroprotective effects in preclinical and limited clinical studies. Mind-body practices like Kirtan Kriya meditation have also demonstrated stress-reduction benefits, addressing modifiable risk factors for AD. While current evidence highlights the potential of TCIM interventions to complement standard care, rigorous validation through high-quality randomized controlled trials remains essential. This review underscores the need for integrative, personalized approaches that synergize traditional and modern medical systems to enhance therapeutic outcomes in AD.

## 1 Introduction

Globally, Alzheimer’s disease (AD) is the primary cause of dementia and cognitive loss in those over 65. Its frequency in this age group varies from 1.9 to 5.8 instances per 100 individuals, and rates rise significantly with age, especially in females (Atri, 2019) (Rocca et al., 1986). Alzheimer’s disease is caused by tau tangles, Aβ plaques, and neuroinflammation, all contributing to the disease (Kabra, 2022; Webers et al., 2020). Aβ aggregation influences tau hyperphosphorylation and microglial inflammation, linking these clinical hallmarks (Kabra, 2022). Pathophysiological changes that characterize this progressive neurodegenerative disease include amyloid-beta plaque buildup, hyperphosphorylated tau protein neurofibrillary tangles, and neurodegeneration due to inflammation and microglial activation (Jack et al., 2013; Li et al., 2022).

In its clinical form, AD first appears as cognitive decline and memory loss, which progresses to neuropsychiatric symptoms including mood swings, disorientation, and severe hallucinations (Harada et al., 2013). Traditional, Complementary, and Integrative Medicine (TCIM) may offer therapeutic alternatives for AD. Mind-body therapy and lifestyle treatments are examples of TCIM techniques that report the multidimensional aspect of AD and complement the limited pharmacological choices available (Nguyen et al., 2024). However, despite the increased interest in TCIM for AD, the number of existing studies and the quality of research are still quite limited. Most research is discredited due to inadequate procedures, such as the lack of strong statistical analysis or RCTs. For this reason, very intensive assessment and cross-disciplinary research must be conducted for TCIM methodologies to be validated and integrated with conventional treatments (Kong et al., 2023). Glutamate antagonists and cholinesterase inhibitors are two examples of current treatments for Alzheimer’s disease that target symptoms rather than the fundamental causes of the illness. This restriction emphasizes how urgently therapies that focus on the intricate pathophysiological pathways of AD are needed (Miculas et al., 2022). TCIM is emphasized in treating Alzheimer’s disease because it considers the human person as a totality-the environmental, behavioral, emotional, and physical factors all contribute to total wellness. More compelling arguments include the less-than-desirable effects of present-day pharmaceutical therapy, the harmful effects they often incur, and further factors. With the complicated nature of AD, this all-embracing approach makes TCIM a useful additional care and preventive approach (Nguyen et al., 2024). The incidence of AD is predicted to triple by 2050, impacting one in every 85 individuals, making it a rapidly expanding worldwide health concern (Brookmeyer et al., 2007). With direct medical expenses varying by country and disease severity, Alzheimer’s disease has a significant financial impact (Takizawa et al., 2014).

Although studies frequently have methodological issues, such as small sample sizes, a lack of controls, and inconsistent results, TCIM shows promise for treating AD. Another issue is publication bias, which favors favorable outcomes while keeping negative discoveries unreported. Extensive, multi-center RCTs are necessary to validate the safety and effectiveness of TCIM. While numerous reviews have explored the potential of Traditional, Complementary, and Integrative Medicine (TCIM) in Alzheimer’s disease (AD), the present manuscript addresses a critical gap by focusing on the multi-targeted and synergistic therapeutic mechanisms of TCIM interventions. Unlike monotherapeutic strategies that often target a single pathological hallmark of AD, TCIM modalities offer a unique advantage through their ability to modulate multiple pathways simultaneously, including oxidative stress, neuroinflammation, mitochondrial dysfunction, and cholinergic deficits. This review consolidates current evidence on the pleiotropic effects of TCIM interventions, offering a systems-level perspective that has not been comprehensively synthesized before. In doing so, we aim to provide a nuanced understanding of how TCIM-based multi-target strategies could complement conventional treatments and improve therapeutic outcomes.

## 2 Methodology

### 2.1 Search strategy

A comprehensive literature search was conducted across PubMed, Scopus, and Web of Science databases from inception to December 2024. The search strategy included combinations of keywords and MeSH terms such as “Alzheimer’s Disease,” “Traditional Medicine,” “Complementary and Integrative Medicine (TCIM),” “Herbal Therapies,” “Neuroinflammation,” “Cognitive Decline,” and “Botanical Drugs.” The Boolean operators “AND” and “OR” were used to refine and expand the search results.

### 2.2 Inclusion and exclusion criteria

To preserve the review’s quality and applicability, stringent inclusion and exclusion criteria were used. Clinical or preclinical results about neuroprotection, anti-inflammatory qualities, and cognitive function improvement were specifically highlighted in studies that examined the therapeutic potential of TCIM in Alzheimer’s disease. Additionally, articles that addressed how TCIM addresses fundamental pathogenic pathways, including amyloid-beta pathology and oxidative stress, were given priority.

On the other hand, research that was unrelated to Alzheimer’s disease or published in a language other than English was disqualified. Furthermore, papers that were only abstracts, editorials, or comments, or those that were not available in full text, were not included. This guaranteed that only comprehensive, peer-reviewed research publications were included in the study.

## 3 Pathogenic pathways in Alzheimer's disease

**FIGURE 1:**
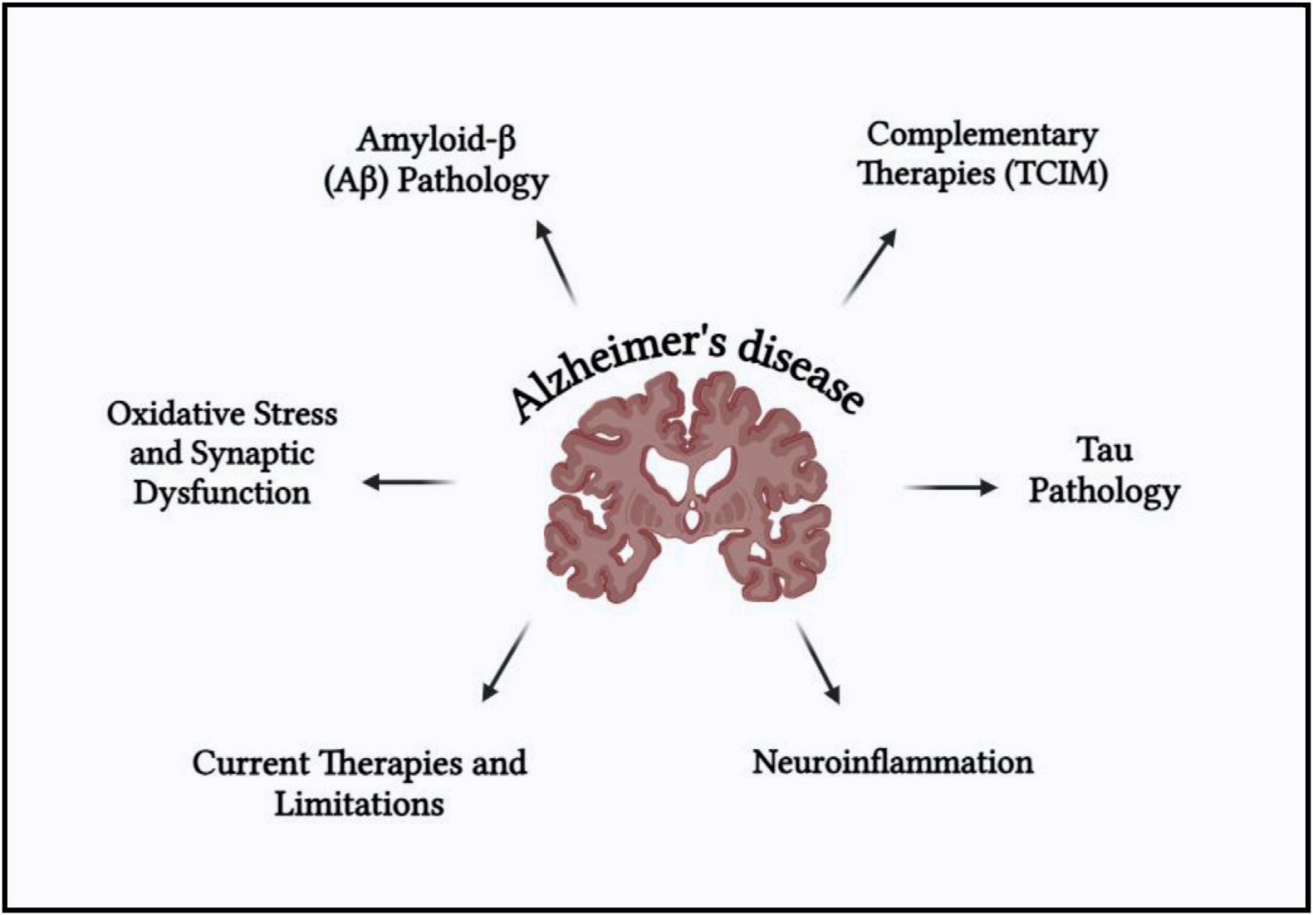
Schematic representation of the multifactorial pathogenesis of Alzheimer’s disease (AD), highlighting key neurobiological mechanisms.

Alzheimer’s disease involves a complex interplay of multiple pathological mechanisms including amyloid-beta (Aβ) accumulation, tau protein hyperphosphorylation, oxidative stress, mitochondrial dysfunction, neuroinflammation, and cholinergic deficits. These interconnected pathways contribute to progressive synaptic failure and cognitive decline. A schematic overview of these multifactorial pathogenic mechanisms is presented in Figure 1.

## 4 TCIM approaches for AD

### 4.1 Foundations and therapeutic principles of TCIM

TCIM, which refers to traditional, complementary, and integrative medicine, is are health practice not very commonly applied by mainstream medicine, but rather serves to supplement the conventional ways of doing things. WHO describes the collective knowledge, skills, and practices acquired from culture and used for the discovery, treatment, and prevention of illness and health-related problems as traditional medicine (Ng et al., 2024). According to the WHO, complementary medicine refers to medical procedures that are utilized in addition to traditional medicine but are not entirely included in the prevailing healthcare system (Ventola, 2010). The term “integrative health,” which refers to the systematic blending of complementary and conventional treatments for patient-centered care, is further expanded by the National Center for Complementary and Integrative Health (NCCIH) (Adib-Hajbaghery et al., 2021). By combining conventional medical procedures with complementary therapies including acupuncture, botanical drugs (e.g., phytochemicals with known antioxidant or anti-inflammatory effects), and to a lesser extent, practices like yoga or meditation that may influence neuroendocrine or oxidative stress pathways. Activities like Kirtan Kriya, a meditation technique that incorporates hand gestures, chanting, and concentrated attention, have demonstrated the potential to lower stress, which is an acknowledged risk factor for AD (Ashford et al., 2015). New studies further reveal that TCIM can be used for the treatment of biological factors involved in AD, such as inflammation and insulin resistance. TCIM thus provides a hopeful scope for management and prevention, yet its validity, safety, and assimilation into conventional practice have to be proved by deeper exploration (Phutrakool and Pongpirul, 2022).

### 4.2 Botanical drugs and phytochemicals

#### 4.2.1 Botanical drugs overview

The three oldest and most vigorous active medical practices are traditional Chinese medicine, traditional Indian medicine, and Ayurveda. The two traditions have sound empirical, experimental, and philosophical evidence. Alternative and complementary medicine have regained popularity for many reasons, such as growing side effects, microbial resistance, emergent diseases, new medications’ high costs, and no efficient treatments for chronic diseases for which no cure is known (Schaffner, 2001). Over 1,500 botanical drugs sold as supplements were among the alternative medicines that two-thirds of Americans were predicted to utilize by 2010 (Legal Status of Traditional Medicine, 2001). With projects in China, Singapore, and Europe, pharmaceutical companies worldwide concentrate on natural product medication development, effectively globalizing Traditional Chinese Medicine (TCM). India’s Ayurveda is also becoming more popular worldwide thanks to government initiatives. According to the Western scientific community, despite their increasing popularity, traditional medicines need scientific confirmation and high-quality research to gain worldwide recognition (Patwardhan and Vaidya, 2014; Patwardhan et al., 2005). *In vitro* models, which lack the intricacy of genuine AD pathophysiology, are used in many TCIM investigations. Although botanical drugs may exhibit anti-inflammatory properties in computational models or cell cultures, their practical effectiveness is still unknown in the absence of *in vivo* validation or clinical studies. Human trials with appropriate controls and well-planned animal experiments should be given top priority in future research.

#### 4.2.2 Specific botanical drugs

Although they are becoming ever more popular, most herbal treatments still do not have concrete scientific evidence. Traditional use oftentimes relies upon empirical use or centuries of experience based on anecdotes, while Western biologic systems require evidence from systematic clinical experience and RCTs. As an example:

Although it is used extensively to enhance memory, in AD research, ginkgo biloba had contradictory effects (Xie et al., 2022). There has been little or uncontrolled investigation of the potential cognitive benefit of Bacopa monnieri, a leading Ayurvedic herb [Bacopa_monnieri_UPDATE_(supplements)]. While curcumin, the active ingredient of turmeric, possesses anti-inflammatory and antioxidant activity, its clinical bioavailability remains challenging (Lopresti, 2018). The disconnect between ancient claims and modern pharmaceutical standards is illustrated by these scenarios, where safety, dose control, repeatability, and bioavailability must be taken into careful consideration (Barnes, 2003).

#### 4.2.3 Mechanisms of action

Several herbal compounds demonstrate interesting biological activity in early-stage investigations, yet much of the data is from *in vitro* studies that fail to convey the sophistication of AD pathology. Many of the studies of TCIM are based on cell models that greatly simplify processes such as amyloid deposition, oxidative stress, and neuroinflammation (shortt et al., 2017).

For instance, it is known to be observed that Icariin inhibits tau phosphorylation and Aβ deposition by modulating the PI3K/Akt/GSK-3β pathway (Wu et al., 2024; Yan et al., 2023).

Ashwagandha and *Centella asiatica* are said to possess anti-inflammatory activity through modulation of cytokines like TNF-α and IL-6, according to studies (Weerasinghe, 2024).

Acetylcholinesterase-inhibiting and able to cross the blood-brain barrier, huperzine A, which is extracted from Chinese club moss, has, in some experiments, been found to bring about symptomatic relief equivalent to that of existing medication, though with fewer side effects (Zhang, 2012).

It is important to validate these pathways using human trials and properly designed *in vitro* animal models. The transition from bench to clinical use has to be bridged in future research to allow for the safe and effective incorporation of candidate herbal therapies into AD therapy.

### 4.3 The role of traditional medicine in Alzheimer’s disease

Although AD is becoming increasingly prevalent across the world and much research is conducted to find a cure, a long-term remedy has yet to be seen. Concerning the devastating loss of cognitive function that patients afflicted with AD experience, powerful therapeutic and preventive treatments are desperately needed to neutralize it. This is very timely as traditional medicine has brought out several promising remedies and drugs for neurodegenerative disorders (Cooper and Ma, 2017). TCM has been practiced for over 2,000 years. TCM or modern pharmacological theories have recently served as the foundation for Chinese botanical drugs used to treat AD; this method has discovered an impact on the causes and mechanisms of AD, TCM treatment, and natural extracts that effectively treat AD. Additionally, there is proof that Chinese botanical drugs may offer some additional cognitive advantages for single-target antagonists (Liu et al., 2014). *Tetradium ruticarpum* (A.Juss.) T.G. Hartley—Family: Rutaceae Bentham is one neuroprotective plant that is frequently used in Traditional Chinese Medicine (TCM) as a herbal remedy. Evodiamine, an extract of *E. rutaecarpa* Bentham, has a wide range of advantageous qualities, including anti-inflammatory and anti-AD roles, as well as antiobesity, antinociceptive, anticancer, antinomic, and antimetastatic effects, all of which are extremely beneficial for treating neurodegenerative diseases (Liu et al., 2013). Additionally, TCM has provided us with substances that need to be improved or substituted for the current AD. Targetin, an agonist of the retinoid X receptor (RXR), effectively treats AD in mouse models. Strong contenders for RXR agonists include the TCM substances sulfonic acid and β-lipoic acid. These TCM chemicals show promise for becoming anti-dementia medications by forming strong interactions via the RXR protein receptors (Chen et al., 2014). The citrus flavonoid nobiletin, which is used in Kampo, or Traditional Japanese Medicine, may be a natural anti-dementia treatment, according to research. Despite these results, the majority of the data supporting Kampo and TCM’s cognitive advantages comes from preclinical or small-scale research. These claims are limited in their robustness by methodological errors, a dearth of placebo-controlled studies, and variations in herbal composition. It has been demonstrated to improve memory and learning, reduce oxidative stress, combat neurodegeneration, encourage synaptic plasticity, and stop plaque development. In a similar vein, ninjin’yoeito, another Kampo drug, has been investigated for enhancing mood and cognitive abilities in Alzheimer’s patients. Depression was decreased, and cognitive function was enhanced when both therapies were combined (Cooper and Ma, 2017). *Centella asiatica* (L.) Urb. — Family: Apiaceae, a plant used as a brain tonic, has been shown to have neuroprotective benefits such as decreasing oxidative stress, inhibiting enzymes, and stopping amyloid plaque formation in AD. Ayurveda, a traditional Indian medicine, has been discovered to include valuable substances for treating problems with the nervous system, such as memory-related conditions like dementia (Nishteswar et al., 2014). Key pathogenic processes in Alzheimer’s disease, such as Aβ aggregation and enzymes involved in the generation of Aβ peptide, can be inhibited by bioactive metabolites found in Traditional Chinese Medicine (TCM). This realization emphasizes the necessity of further research into TCM-derived plant metabolites to identify new mechanisms and synergistic pathways that conventional treatments may influence. Certain TCM elements can reduce inflammation and neuronal cytotoxicity brought on by Aβ. The review raises the idea of using TCM to treat AD and the opportunity for further TCM ingredient screening for therapeutic uses (Cheung et al., 2015). AD may benefit from the antioxidant and anti-apoptotic properties of several herbal medications and phytochemicals. Herbal medications and substances have been shown in preclinical investigations in animal models of AD to have antioxidant properties, enhance cognition, and offer neuroprotection. However, drug-herb interactions, dosage consistency, and long-term effectiveness must all be carefully considered when combining conventional therapy with contemporary pharmaceutical treatments. Guidelines based on evidence would aid in bridging the gap between conventional medicine. According to traditional wisdom, herbal treatments may be able to address the pathophysiology of AD at several molecular and cellular levels (Wadhawan et al., 2024). Medicinal plants, such as *Tinospora cordifolia* (Willd.) Miers—Family: Menispermaceae, *Hericium erinaceus* (Bull.) Pers. — Family: Hericiaceae, *Commiphora wightii* (Arn.) Bhandari—Family: Burseraceae, *Hypericum perforatum* L. — Family: Hypericaceae, *Convolvulus pluricaulis* Choisy—Family: Convolvulaceae, *Rhodiola rosea* L. — Family: Crassulaceae, *Moringa oleifera Lam*. — Family: Moringaceae, and *Camellia sinensis* (L) Kuntze—Family: Theaceae are a few examples of medicinal plants that have both preventative and therapeutic effects on AD. Information from observational research is scarce (Perry et al., 1999; Sutalangka et al., 2013; Zieneldien et al., 2022). Although Randomized controlled trials and well-designed longitudinal investigations are required to determine the effectiveness, safety, and therapeutic range of traditional herbal treatments, to expand the body of evidence.

• *Ginkgo biloba* L. — Family: Ginkgoaceae, a well-studied herbal extract in the context of neurodegenerative diseases, has shown promising effects in various experimental models. *In vitro* studies reveal that it exerts antioxidant and neuroprotective effects by protecting neurons from oxidative stress and β-amyloid-induced cytotoxicity. *In vivo* findings in animal models of Alzheimer’s disease demonstrate improved cognitive performance, enhanced cerebral blood flow, and restored mitochondrial function. However, while some *clinical trials* have reported improved memory and cognitive scores, particularly with long-term administration, the overall quality of evidence remains mixed. Meta-analyses indicate heterogeneity in outcomes, underscoring the need for more robust, large-scale randomized controlled trials (RCTs) (Yang et al., 2015).
• Huperzine A, an alkaloid derived from *Huperzia serrata* (Thunb.) Trevis. — Family: Lycopodiaceae, has been identified as a potent reversible inhibitor of acetylcholinesterase (AChE). *In vitro*, it shows neuroprotective activity against glutamate-induced excitotoxicity and oxidative injury. *In vivo* studies report enhancements in memory retention, learning behavior, and synaptic density in rodent models of Alzheimer’s disease. In *clinical settings*, small-scale RCTs have demonstrated cognitive improvements in individuals with Alzheimer’s and vascular dementia, with favorable tolerability. Despite encouraging findings, caution is warranted when interpreting these results due to limitations in trial design and sample size (Rafii et al., 2011).
• *Centella asiatica* (L.) Urb. — Family: Apiaceae has long been used in traditional medicine for cognitive support. Its *in vitro* profile includes promotion of neurite outgrowth, enhanced collagen synthesis, and modulation of antioxidant enzymes. In *in vivo* models, it has been shown to improve spatial learning and memory, increase hippocampal dendritic arborization, and support synaptic plasticity. Preliminary *clinical evidence* supports its potential, particularly in elderly populations where improvements in mood and memory have been observed. Nonetheless, these results require validation through well-powered clinical studies (Wright et al., 2022).
• *Curcuma longa* L. — Family: Zingiberaceae, the principal curcuminoid of *C. longa*, exhibits a diverse range of bioactivities. *In vitro*, it demonstrates antioxidant, anti-inflammatory, and anti-amyloidogenic effects by modulating signaling pathways like NF-κB, Nrf2, and β-secretase activity. *In vivo* animal models show that *C. longa* L. — Family: Zingiberaceae can reduce amyloid plaque burden, promote neurogenesis, and enhance memory performance. However, *clinical trials* have yielded inconsistent outcomes, largely due to curcumin’s poor oral bioavailability. While some trials suggest modest cognitive benefits, further studies employing bioavailability-enhanced formulations are necessary (Kuszewski et al., 2018).
• *Withania somnifera* (L.) Dunal—Family: Solanaceae has demonstrated promising neuroprotective effects across multiple experimental levels. *In vitro*, it stimulates neurite outgrowth, suppresses oxidative stress, and downregulates inflammatory mediators. *In vivo* studies reveal its ability to reverse cognitive deficits in stress-induced and transgenic mouse models of Alzheimer’s disease, with noted improvements in learning and memory. Clinical investigations have also shown favorable results in improving attention, executive function, and information processing speed in both cognitively impaired and healthy adults. Despite its traditional use and growing scientific support, large-scale, blinded RCTs are still needed to substantiate its clinical efficacy in dementia care (Xing et al., 2022)

### 4.4 Complementary and integrative medicine

The National Center for Complementary and Integrative Health (NCCIH) classifies TCIM approaches into three categories (a) mind-body practices (e.g., yoga, meditation, acupuncture): that may exert effects through modulation of stress hormones like cortisol or neurotrophic factors such as BDNF; (b) traditional medical systems such as Ayurveda and Traditional Chinese Medicine (TCM), which offer pharmacologically active herbal agents; and (c) natural products, including botanical medicines, vitamins, and probiotics with potential neuroprotective or anti-inflammatory properties (Nguyen et al., 2022).

It has been shown that using these adjunct methods along with drug treatments results in synergistic outcomes. For example, in AD patients, donepezil and yoga or mindfulness-based stress reduction have been associated with improved mood, reduced anxiety, and enhanced cognitive functioning. Cholinesterase inhibitors also seem to facilitate neuroprotection and delay cognitive decline when used along with nutritional therapies like the Mediterranean diet. These combined strategies illustrate how an integrated, multimodal approach could enhance overall outcomes of Alzheimer’s disease treatment (García-Casares et al., 2021).

Although preclinical and clinical research have shown encouraging benefits, there is still conflicting evidence in favor of complementary and integrative therapies for AD. Thorough clinical studies with specified objectives are required to confirm their effectiveness and clarify the exact processes behind their possible advantages.

### 4.5 Modulation of the microbiota-gut-brain axis in Alzheimer’s disease through TCIM approaches

The microbiota-gut-brain axis includes bidirectional communication via neuroimmune, neuroendocrine, and direct neural routes, such as the vagus nerve (Peterson, 2020). The condition contributes to AD pathology by impairing host immunological responses and increasing inflammation, potentially acting as a trigger for the start and development of AD. TCM is a viable resource for treating Alzheimer’s disease because of its chemical diversity and multi-target properties. It modulates the gut microbiota, which is a key target for its ability to cure Alzheimer’s disease by modifying the microbiota-gut-brain axis (Ma et al., 2023).

Traditional Chinese medicine can prevent and treat Alzheimer’s disease by regulating the gut microbiota (Long et al., 2024).

Mechanism: Changes in the gut plants may contribute to the buildup of amyloid beta. The relationship between the gut and the brain, and metabolites produced by the gastrointestinal microbiome, is involved in Alzheimer’s disease pathogenesis.

Clinical trial evidence: Recent preclinical and clinical research suggests that gut microbiota changes may play a role in Alzheimer’s disease. Antibiotics, prebiotics, probiotics, fecal microbiota transplantation, and dietary changes are all possible treatments.

Emphasizes the efficacy of antibiotics, prebiotics, probiotics, feces microbe transplantation, and dietary changes as complementary therapy for Alzheimer’s disease, demonstrating their incorporation in mainstream pharmacological practice (Guo et al., 2020).

## 5 Mechanisms of action in TCIM

**FIGURE 2:**
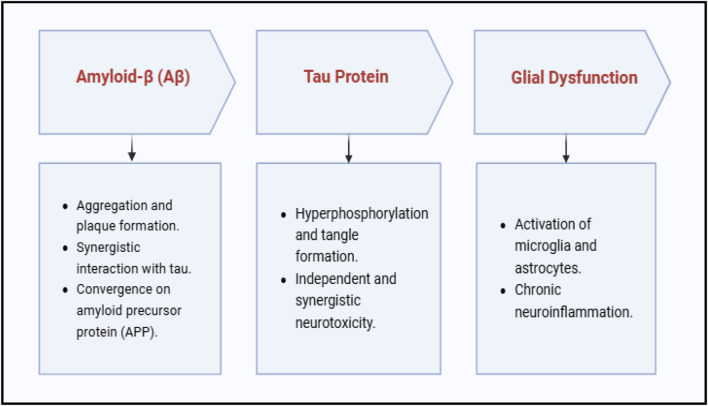
Overview of molecular and cellular mechanisms modulated by selected TCIM interventions.

Amyloid β, tau, and glial dysfunction are the main contributors to the growth of AD (Han et al., 2020). Key Contributors and Therapeutic Pathways in AD are illustrated in Figure 2.

*Curcuma longa* L. — Family: Zingiberaceae has been explored as a treatment that influences pathways such as PI3K/Akt signaling and neurotrophin signaling (Bashir et al., 2022). TMS has been shown to promote synaptic plasticity, change gene expression, and boost cognitive function in AD patients (Zhao et al., 2019). In AD mouse models, trans-cinnamaldehyde (TCA) has also been demonstrated to have neuroprotective effects via improving NMDA receptor activity and decreasing neuroinflammation. TCA lowers pro-inflammatory mediator levels and microglial activation by blocking the NF-κB pathway (Prasad et al., 2002). AD is a neurodegenerative condition complicated by oxidative stress, neuroinflammation, and loss of cognitive functions (Papagiouvannis et al., 2021) (Murphy and Park, 2017). Studies indicate that treatment of AD is possibly more effective using the multi-target approach, such as including antioxidants, anti-inflammatory drugs, and neurotrophic factors, rather than single-modal therapy (Papagiouvannis et al., 2021) (Onaolapo et al., 2021).

Activating the Nrf2-mediated antioxidant defense system and stimulating the neurotrophic signaling pathway can potentially change AD pathology (Onaolapo et al., 2021). Additionally, nutritional treatment and lifestyle modifications may be crucial in lowering the oxidative stress and neuroinflammation associated with Alzheimer’s. Though preclinical studies have shown that anti-inflammatory medicines are beneficial, this finding has yet to be confirmed in clinical investigations, necessitating additional studies into more viable treatment strategies (Ghosh et al., 2013). Tau immunotherapy can regulate both tau and upstream amyloid pathology, while persistent interleukin-1β overexpression worsens tau pathology despite reduced amyloid burden (Castillo-Carranza et al., 2015) (Haut et al., 2023). The “Amyloid Cascade Hypothesis,” which holds that tau and Aβ separately produce neurotoxicity, is called into question by these findings (Busche and Hyman, 2020) Research shows that tau and Aβ work in concert, convergently acting on the amyloid precursor protein (APP) as a common downstream effector (Busche and Hyman, 2020) (Wang C. et al., 2014). This emphasizes the necessity of concurrently focusing efforts on both (Wang C. et al., 2014). It has been shown in traditional medicine that *C. longa*, or *C. longa* L. — Family: Zingiberaceae, activates the PI3K/Akt pathway, increasing neuronal survival and lowering damage brought on by Aβ (Limpeanchob et al., 2008). *Bacopa monnieri* (L.) Wettst. — Family: Plantaginaceae also improves Nrf2 pathway activity, which reduces oxidative stress and increases cellular resilience (Dhanasekaran et al., 2009). According to recent research, *Centella asiatica* (L.) Urb. — Family: Apiaceae extracts considerably lower oxidative stress indicators and hippocampus Aβ levels in AD mouse models, confirming its function in protective effects on neurons and cognitive improvement (Jayaprakasam et al., 2010). Clinical investigations verifying these cognitive advantages are still few, despite preclinical research suggesting *C. asiatica* (L.) Urb. — Family: Apiaceae may have a neuroprotective function. Furthermore, *in vivo* and clinical studies have demonstrated that *Withania somnifera* reduces tau aggregation and enhances antioxidant defense (Kuboyama et al., 2002; Li et al., 2008). TCIM drugs like Fuzhisan that increase acetylcholine content with additional anti-inflammatory and antioxidant activities share the same aim of achieving and maintaining adequate acetylcholine levels that conventional therapy, including cholinesterase inhibitors, aims to achieve. Because TCIM mechanisms can target various pathways implicated in AD pathogenesis, they can complement traditional therapies, which highlights the promise of integrative therapy methods (King et al., 2020). However, the absence of established procedures, dosage fluctuations, and a dearth of solid clinical data pose serious obstacles to the broader use of TCIM. Evidence-based studies that align TCIM techniques with traditional medical procedures may offer the necessary scientific support for their broad application.

TCIM-based therapies have tremendous promise for the treatment of Alzheimer’s disease, as they can target most of the pathogenic pathways, such as oxidative stress, inflammation, tau pathology, and cholinergic deficits. Overcoming critical obstacles such as bioavailability, pharmacokinetics, and herbal metabolite standardization will, however, need to be overcome for preclinical success to translate to clinical application. Integrative techniques that combine TCIM with conventional medicines have the potential to provide synergistic advantages in AD management.

### 5.1 Pharmacological and clinical validation of herb-derived agents in Alzheimer’s disease

#### 5.1.1 Pharmacological mechanisms of action

Herb-derived phytochemicals target multiple pathogenic pathways in AD (Table 1). The pharmacological mechanisms of key herbal agents in AD are summarized in Table 1.

**TABLE 1:** Pharmacological mechanisms of action of selected herb-derived agents in Alzheimer’s disease.

Herb	Absorption	Metabolism	BBB penetration	Half-life
*Curcuma longa* L. — Family: Zingiberaceae	Poor (<1%)	Extensive hepatic glucuronidation	Low	∼1–2 h (Gupta et al., 2022)
*Withania somnifera* (L.) Dunal — Family: Solanaceae	Moderate	Hepatic (CYP3A4)	Moderate	∼6 h (Aguiar and Borowski, 2013)
Bacopa	Variable	Hepatic	Moderate	∼4–6 h (Zullino et al., 2003)
Ginkgo	Good	CYP2C19, 2D6 interaction	Yes	∼4–6 h (Diniz et al., 2023)
Centella	Low	Liver via phase I/II	Moderate	∼3–5 h (Patwardhan et al., 2005)

*Curcuma longa* L. — Family: Zingiberaceae: Inhibits Aβ aggregation, activates PI3K/Akt pathway, and downregulates NF-κB-mediated neuroinflammation. [IC50 for AChE inhibition: ∼67.69 µM] (Sehgal et al., 2012).

*Withania somnifera* (L.) Dunal—Family: Solanaceae: Inhibits acetylcholinesterase, reduces TNF-α and IL-6 levels, and promotes neurogenesis via LRP1 receptor upregulation (Huang et al., 2008).

*Bacopa monnieri* (L.) Wettst. — Family: Plantaginaceae: Enhances BDNF expression and reduces oxidative stress through upregulation of antioxidant enzymes (SOD, catalase) (Yang et al., 2015).

*Ginkgo biloba* L. — Family: Ginkgoaceae: Reduces tau hyperphosphorylation, enhances cerebral blood flow, and exerts antioxidant effects (Yin et al., 2009).

*Centella asiatica* (L.) Urb. — Family: Apiaceae: Protects neurons via antioxidant activity and modulates MAPK/ERK and PI3K/Akt signaling pathways (Anand et al., 2007).

#### 5.1.2 Pharmacokinetics and pharmacodynamics (PK/PD)

Despite promising effects, botanical drugs face PK/PD challenges: Table 2 outlines the absorption, metabolism, BBB penetration, and half-life of commonly used herbs in AD.

**TABLE 2:** Pharmacokinetic properties of key herbal drugs used in Alzheimer’s disease.

Herb	Affected drug	Mechanism	Clinical concern
Ginkgo	Donepezil	CYP2D6/3A4 modulation	Altered plasma levels
*Curcuma longa* L. — Family: Zingiberaceae	Rivastigmine	Inhibits P-gp	Potential toxicity (Shoukat et al., 2023)
Bacopa	Galantamine	Additive AChE inhibition	Cholinergic excess risk (Candelario et al., 2015)
*Withania somnifera* (L.) Dunal — Family: Solanaceae	Benzodiazepines	GABAergic potentiation	Excess sedation (Small et al., 2018)

#### 5.1.3 Quality control aspects

Standardization and quality assurance of botanical drugs remain significant concerns in TCIM. Phytochemical content variation can arise due to environmental factors like climate, harvesting season, and post-harvest storage conditions (Saper et al., 2008). This inconsistency affects therapeutic efficacy and reproducibility. Additionally, herbal products may be contaminated with pesticides, heavy metals, or microbial agents, posing potential health risks (WHO, 2024). Regulatory agencies, including the World Health Organization (WHO), advocate for the implementation of Good Agricultural and Collection Practices (GACP) and standardized manufacturing methods such as High-Performance Liquid Chromatography (HPLC) to ensure quality and safety (Kunle, 2012). Identification and quantification of specific marker metabolites, such as withanolides in *Withania somnifera* (L.) Dunal—Family: Solanaceae, bacosides in *Bacopa monnieri* (L.) Wettst. — Family: Plantaginaceae, and the standardized extract EGb761 in *Ginkgo biloba* L. — Family: Ginkgoaceae, are essential for product consistency and efficacy validation (Flory et al., 2021).

#### 5.1.4 Herb-drug interactions

Several herb-drug interactions can affect conventional AD treatments. Common herb-drug interactions that may influence pharmacotherapy in AD are detailed in Table 3.

**TABLE 3:** Herb-drug interactions relevant to conventional Alzheimer’s disease therapies.

Agent	Study design	Sample size	Outcome	Limitations
*Curcuma longa* L. — Family: Zingiberaceae	RCT	n = 132	Reduced inflammatory markers; no cognitive improvement	Poor bioavailability (Choudhary et al., 2017)
*Withania somnifera* (L.) Dunal — Family: Solanaceae	RCT	n = 50	Memory improvement (MMSE)	Small sample, short duration (Yang et al., 2015)
Ginkgo	Meta-analysis	21 trials, n > 2600	Mild cognitive benefit (MMSE, ADL)	Heterogeneous studies (Hutchison and Rodgers, 2001)
Bacopa	RCT	n = 98	Improved memory retention	Variability in extracts (Pullar et al., 2018)
Centella	reclinical	n/a	Reduced Aβ burden in mice	Limited human data (Article-034.).

#### 5.1.5 Clinical evidence and efficacy evaluation

Several human studies have explored herbal efficacy, but limitations remain.

## 6 Enhanced therapeutic potential of herbal combinations

**FIGURE 3:**
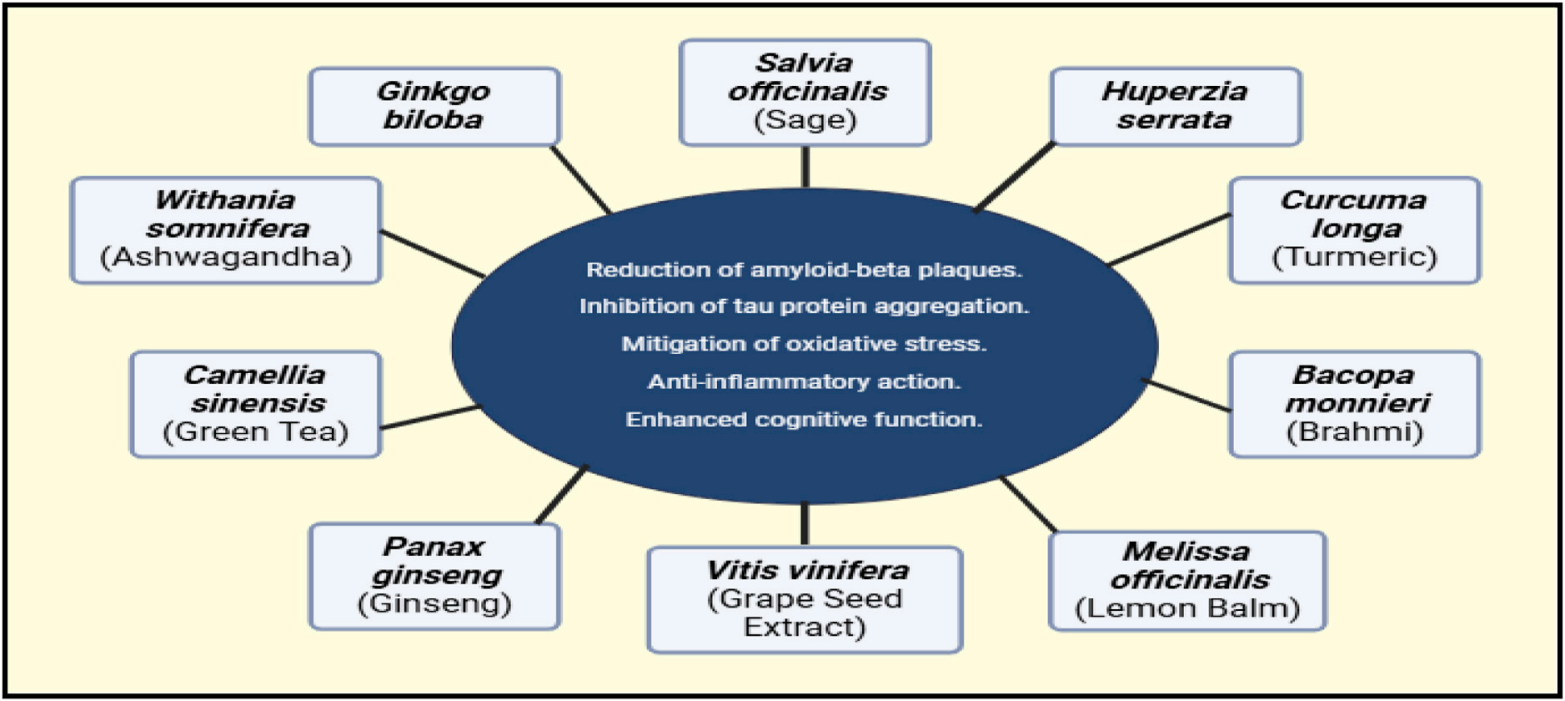
Proposed synergistic effects of various TCIM interventions in AD. This figure is based on theoretical predictions derived from existing literature and mechanistic insights, rather than direct experimental evidence.

Multi-metabolite botanical drugs are widely recognized for potential use in treating complex diseases such as AD. These alternatives have several key advantages over more conventional treatments, among which are improved safety profiles, relative cost-effectiveness, and potential multi-target efficacy. Traditional Oriental herbal formulations have been shown to address multiple aspects of AD by diverse mechanisms (Jeon et al., 2019). Illustrations of Synergistic Properties of Multi-metabolite Herbal Therapies in Figure 3.

The multi-metabolite nature of herbal preparations and the existence of unknown plant metabolites pose challenges in metabolism studies. Nevertheless, recent improvements in bioanalytical technologies have improved our capacity to assess the pharmacokinetics and metabolic interactions of plant metabolites (Xin et al., 2011).

These treatments have also been able to counteract some of the chemotherapy-induced toxicities, including neurotoxicity and cardiotoxicity (Fu et al., 2018). The neuropathological factors involved, such as tau, α-synuclein, and β-amyloid (Aβ), are synergistic and interact with each other in a complex manner, different from the classical linear progression model (Clinton et al., 2010). Thus, a single protein cannot be targeted, since the anti-Aβ clinical studies were not successful enough (Wang J. et al., 2014). Promising approaches are multi-target therapies, which include polyphenolic metabolites. Comparison between grape seed extract, resveratrol, and purple grape juice extract showed better outcomes than single metabolite treatment on cognition scores and lowering of amyloid loads in AD models of animal systems (Calfio et al., 2020). It forms a significant problem in producing effective treatments against AD. Recent studies focused on using the possibilities of multi-target treatments of natural substances and phytochemicals, which could serve to mitigate Alzheimer’s disease (Tuzimski and Petruczynik, 2022) (Calfio et al., 2020). Multi-target therapies, including polyphenolic compounds, have emerged as promising strategies. Research comparing grape seed extract, resveratrol, and purple grape juice extract with single-metabolite treatments found improved cognitive outcomes and reduced amyloid load in animal models of AD (Tuzimski and Petruczynik, 2022) (Iqubal et al., 2021). Natural compounds, however, tend to attack the complexities of Alzheimer’s disease from various angles, whereas synthetic drugs tend to target a particular target (Wang J. et al., 2014). Unlike synthetic drugs, which often target a single pathway, natural compounds exhibit a broader therapeutic scope. Plant extracts and isolated phytochemicals are gaining attention as potential AD therapeutics, with many showing promise in preclinical studies (Bhat et al., 2022; Oxford et al., 2020).

## 7 Evaluation of clinical studies and therapeutic efficacy

Recent AD pharmacological trials have focused on beta-amyloid, which has usually failed to enhance cognitive function (Tripathi et al., 2024). The other targets that scientists consider include tau protein, neuroinflammation, and oxidative stress. A summary of clinical investigations evaluating TCIM interventions in Alzheimer’s disease is provided in Table 4. Similarly, Table 5 provides an overview of experimental studies elucidating the mechanisms of action of TCIM approaches in Alzheimer’s disease. Selected landmark clinical trials investigating both conventional and herbal interventions in Alzheimer’s disease are summarized in Table 6. Plant-based traditional remedies like *Ginkgo biloba* L. — Family: Ginkgoaceae, *Huperzia serrata* (Thunb.) Trevis. — Family: Lycopodiaceae, and *Curcuma longa* have been shown to influence these targets through plant metabolites (Lee et al., 2020). Randomized controlled trials have been conducted to look at botanical drug therapies for AD, and Mini-Mental State Checkup scores have been used as a key outcome measure (Gharat et al., 2024). Cholinesterase inhibitors are already in use for the treatment of Alzheimer’s disease symptoms, and there is an urgent need for disease-modifying medication (Cocchiara et al., 2020). Management of AD requires initial diagnosis and proper treatment, encompassing both drug and nondrug therapies (Pimperkhede et al., 2023).

**TABLE 4:** Summary of clinical studies evaluating TCIM in Alzheimer’s disease.

Study design	Sample size and characteristics	Intervention	Outcome measures	Effect size/Results	Statistical significance	Reference
Randomized Controlled Trial (RCT)	72 patients with mild-to-moderate AD	Sailuotong (Ginseng, Ginkgo, Crocus)	MMSE, ADAS-Cog	Improved MMSE scores by 3.2 ± 1.4 points	p < 0.01	Steiner‐Lim et al. (2023)
Double-blind RCT	60 elderly patients diagnosed with AD	Bushen capsule	ADAS-Cog, memory score	Memory scores improved significantly over placebo	p < 0.05	Zhang et al. (2019)
Pilot RCT	34 AD patients (aged 65–85)	Acupuncture (weekly sessions for 12 weeks)	ADAS-Cog, behavior rating	ADAS-Cog improved by 2.7 points on average	Not reported	Lin et al. (2021)
Case-control	40 patients, 40 controls	Tai Chi exercise program (12 weeks)	Cognitive subtests	Delayed decline in executive function	p < 0.05	Yassine and Finch (2020)
Open-label clinical trial	50 patients with mild AD	Ginkgo biloba L. — Family: Ginkgoaceae extract (EGb761)	MMSE, Clock Drawing Test	MMSE scores improved by 2.4 ± 0.9	p < 0.05	Strandberg (2019)

**TABLE 5:** Mechanistic studies on TCIM modalities in Alzheimer’s disease.

Type of study	Model/Sample	Intervention	Outcome/Mechanism	Effect size/Results	Statistical significance	Reference
In vivo (mice)	APP/PS1 transgenic mice	Ginseng extract	Reduced Aβ deposition, improved memory function	40% reduction in Aβ plaques	p < 0.01	Choi et al. (2017)
In vitro	SH-SY5Y neuroblastoma cells	Resveratrol	Inhibited tau phosphorylation and oxidative stress	60% decrease in p-tau levels	p < 0.001	Inoue et al. (2016)
In vivo	Rat model of AD	Acupuncture	Increased BDNF and synaptic plasticity	BDNF upregulated 2.5-fold	p < 0.01	Nakamura and Sugaya (2014)
In vivo	SAMP8 mice	Curcuma longa L. — Family: Zingiberaceae	Anti-inflammatory and antioxidant effects	IL-6 levels decreased by 30%	p < 0.05	Kabak et al. (2021)
In vitro	Astrocyte cell line	Ginkgo biloba L. — Family: Ginkgoaceae extract	Increased mitochondrial viability	ATP production increased by 45%	p < 0.01	Qiu et al. (2022)

**TABLE 6:** Clinical trials related to Alzheimer’s disease.

Trial name/Study	Intervention	Key outcome	Limitations
EXPEDITION-3 Trial	Solanezumab (Anti-Aβ monoclonal antibody)	Failed to enhance cognitive function in mild AD patients (Tripathi et al., 2024)	Targeted only Aβ; ignored tau and other mechanisms
Ginkgo Evaluation of Memory Study (GEMS)	*Ginkgo biloba* L. — Family: Ginkgoaceae extract	There was no significant decrease in the incidence of AD compared to the placebo (Lee et al., 2020)	The study population is mostly elderly with high comorbidities
HUPER Study	Huperzine A	Exhibited moderate improvement in cognition and decreased oxidative stress (Lee et al., 2020)	Small sample size; short trial duration
CURCUMIN Study	*Curcuma longa* L. — Family: Zingiberaceae supplements	Marginal effects on cognitive outcomes, but reduced neuroinflammation markers	Poor bioavailability; variability in *Curcuma longa* L. — Family: Zingiberaceae formulations (Lee et al., 2020)
SYNAPSE Study	Acetylcholinesterase inhibitors (donepezil)	Improved cognitive scores in mild to moderate AD patients (Gharat et al., 2024)	No disease-modifying effects; focused only on symptom management
FIT-AD Study	Aerobic exercise	Significant improvement in cognitive and functional abilities (G et al., 2021)	Lack of standardization in exercise regimens; challenging adherence
CONCORD-AD Network	Multinational cohort studies	Provided insights into the natural history of AD and trial design (Parfenov, 2020)	Heterogeneous populations; limited focus on emerging biomarkers

Acetylcholinesterase inhibitors and memantine are the two most commonly prescribed pharmacotherapies; however, herbal treatments are also being researched (Pavlik et al., 2022). The CONCORD-AD network consists of seven multinational cohorts and aims to provide greater insight into the natural history of AD, thereby informing the design of clinical trials (Parfenov, 2020). However, management mistakes prevail; these include underdiagnosis, misunderstanding contemporary medicines, and the use of anti-dementia drugs. Non-pharmacological interventions like stimulation, exercise, and antioxidant foods are often overlooked (Andrade et al., 2022). The education and support of the caregivers are essential to provide excellent patient care (Pimperkhede et al., 2023). More knowledge among healthcare providers, improved diagnosis techniques, and access to effective medications would be vital in enhancing the management of AD (Andrade et al., 2022) (Pimperkhede et al., 2023). Meta-analyses and systematic reviews in recent times have shown significant insights into the prevention and treatment of AD. Exercise, specifically aerobic and multimodal exercise, has been proven to decrease AD symptoms, most importantly, cognitive (G et al., 2021).

Some gene variations, including CD33, BIN1, and MTHFR, have been identified as contributing to the risk of AD in several studies (Yu et al., 2020). A comprehensive assessment of modifiable factors disclosed 21 evidence-based notions for preventing AD, with great evidence for education, cognitive activity, and health conditions (Ou et al., 2020). Lastly, infectious agents have also been linked to the risk for Alzheimer’s disease, which has been found to increase with many infections, including *Chlamydia* pneumoniae, Human herpesvirus-6, and Herpes simplex virus-1, with significant connections (Kumar et al., 2016). New-generation improvements in diagnostic tools and biomarker-based assessments will even increase trial reliability. Currently, the landscape of AD clinical trials is characterized by narrow target approaches, potential biases, small sample sizes, and variability in interventions. These must be overcome with multi-target therapeutic approaches, long follow-ups, and standardized protocols in drug and non-drug therapy.

## 8 Novel therapeutic targets: advances and emerging strategies

AD is a neurodegenerative illness caused by tau tangles, β-amyloid plaques, and neuroinflammation. Research efforts are directed toward novel targets and multifunctional compounds to address the multifaceted aspect of Alzheimer’s pathogenesis. Computational biology, particularly molecular docking, has emerged as an effective methodology for discovering new therapeutic candidates. Alzheimer’s disease is linked to multiple signaling pathways that have been involved in its etiology. Some key processes are abnormal calcium homeostasis, amyloid-beta plaque formation, and tau protein hyperphosphorylation. The PI3K/AKT pathway regulates cell survival and death, which is important for neuroprotection (Long et al., 2021). Autophagy, which interacts with several AD-related processes, is critical for maintaining cellular homeostasis and removing harmful proteins (Ramachandra et al., 2021).

APOE, GSK-3β, Notch, and Wnt signaling have also recently been associated with AD (Long et al., 2021). Some studies are currently on multi-convergence, with studies suggesting that autophagy serves as a potential therapeutic target, with its critical role and multiple interactions (Gadhave et al., 2021). In addition, natural compounds are identified as activators of PI3K/AKT; hence, they may come into use as neuroprotective drugs in the pharmacotherapy of AD. Current treatments focus on early intervention and a holistic approach to the various disease processes. The targets include amyloid beta, tau proteins, neurotransmission, inflammation, metabolism, and oxidative stress (Hroudová and Fišar, 2024). There are only a few medications that are accepted for the treatment of AD, such as cholinesterase inhibitors, NMDA antagonists, and anti-Aβ monoclonal antibodies (Abdallah, 2024) (Athar et al., 2021). The researchers are working on innovative therapeutic methods such as immunotherapy and neuroinflammation (Abdallah, 2024). An overview of promising therapeutic innovations, including gene therapy, nanotechnology, and neuroregenerative strategies, is summarized in Table 7. Other promising alternative techniques for AD treatment include targeted deep brain stimulation, ultrasound, stem cell therapy, and gene therapy, as represented in Table 4 for Innovative Approaches in AD Management.

**TABLE 7:** Emerging therapeutic**s** in Alzheimer’s disease management.

Technique	Therapeutic target	Advantages	Challenges
Gene Therapy	Amyloidβ, Tau protein	Potential to modify disease progression	High cost, ethical concerns
Targeted Ultrasound	Amyloid Plaques	Non-invasive, improves drug delivery	Needs advanced equipment and training
Deep Brain Stimulation	Neurotransmission	Improves cognitive functions	Invasive procedure, potential side effects
Stem cell therapy	Neuroregeneration	Promotes neuronal repair	In a few clinical trials, the potential for oncogenesis
Immunotherapy	Amyloid β, Tau Proteins	Decreases pathological aggregates	Limited clinical trials, risk of tumorigenesis
Nanotechnology	Drug Delivery Systems	Increases blood-brain barrier permeability	Difficult manufacturing

Yet, there is still the issue of presymptomatic neuronal injury, undesirable effects of medication, and poor clinical trial design (Tatulian, 2022). Future research may be focused on AD pathophysiology, biomarkers, and new diagnostic tools, thus allowing for the development of more effective therapy strategies in the future (Hroudová and Fišar, 2024) (Tatulian, 2022). The limited absorption and quick metabolism of botanical drugs such as *Curcuma longa* L. — Family: Zingiberaceae and *Bacopa monnieri* (L.) Wettst. — Family: Plantaginaceae makes it difficult to translate preclinical findings of TCIM treatments for AD into human trials. Variability in extract quality and irregular dosage further restricts reproducibility. The multi-target mechanisms by which TCIM interventions modulate Alzheimer’s pathology are illustrated in Table 8. Animal models often cannot mimic the intricate pathology of Alzheimer’s disease observed in humans, which adds to discrepancies between preclinical and clinical outcomes. In addition, short-term follow-up studies with smaller sample sizes limit the capacity of the trials to reveal important disease-modifying benefits. In addition, there are no standard procedures followed, and only a few biomarkers are used, hence, it is impossible to replicate reproducible results. Future studies would be more optimized in drug delivery systems, and further standardization of formulations can be done using advanced biomarkers and lengthier studies to decide the therapeutic efficacy of TCIM.

**TABLE 8:** Mechanism of TCIM in Alzheimer’s disease.

Therapeutic agent	Pathways targeted	Mechanism of action	Study design	Reference
*Curcuma longa* L. — Family: Zingiberaceae	PI3K/Akt Signaling	Promotes neuronal survival, reduces Aβ-induced damage	Preclinical models show reduced Aβ plaque levels	Bashir et al. (2022)
*Bacopa monnier*i (L.) Wettst. — Family: Plantaginaceae	Nrf2 Antioxidant Pathway	Enhances antioxidant defenses, mitigates oxidative stress	Preclinical studies support cognitive improvement	Dhanasekaran et al. (2009)
*Centella asiatica* (L.) Urb. — Family: Apiaceae	Oxidative Stress and Aβ Pathways	Reduces hippocampal Aβ levels and oxidative stress markers; improves cognitive function	Evidence in AD mouse models	Jayaprakasam et al. (2010)
*Withania somnifera*	Tau Aggregation and Antioxidant Défense	Reduces tau aggregation, enhances antioxidant defenses	Supported by *in vivo* and clinical studies	Kuboyama et al. (2002), Li et al. (2008)
Fuzhisan	Cholinergic Pathway, Antioxidants, Anti-Inflammatory	Increases acetylcholine levels, provides anti-inflammatory and antioxidant benefits	Clinical trials indicate cognitive enhancement	King et al. (2020)

## 9 Innovation and future directions

There are numerous limits to current Alzheimer’s disease research. While artificial intelligence and language processing show potential for detecting cognitive decline, low standardization and restricted result comparability impede the clinical application (de la Fuente Garcia et al., 2020). Existing pharmacological treatments only provide symptomatic alleviation, while disease-modifying medicines remain unavailable. Central nervous system drug delivery is still challenging. However, nanoliposomes and exosomes have the potential to improve brain function (Passeri et al., 2022). The preclinical animal models are poor at simulating human AD pathology, leading to a poor transformation of hopeful applicants into clinical trials (Akhtar et al., 2022). Neuroimaging techniques have emerged as promising tools for the early diagnosis of Alzheimer’s disease and the identification of biomarkers. However, their clinical utility varies in terms of precision and effectiveness (Kim et al., 2022).

Future drugs will focus on the fundamental mechanisms of diseases like Aβ plaques and neurofibrillary tangles, besides clinical relief (Yiannopoulou and Papageorgiou, 2020). Biomarkers of Aβ, tau pathology, neurodegeneration, synaptic dysfunction, and inflammation are important for selecting the right people to be studied and assessing the degree of their improvement (Zetterberg and Bendlin, 2021). The growing interest in psychedelic therapy for Alzheimer’s disease raises ethical concerns, for example, about the effect on autonomy, consent, and caregiving, not to mention the potential exploitation of desperation in patients (Peterson et al., 2023). As science moves forward toward personalized medicine, various biomarkers combined with cognitive and neuroimaging data might guide treatment regimens (Yiannopoulou and Papageorgiou, 2020). In preparing healthcare systems for disease-modifying medications and addressing the ethical concerns surrounding psychedelic research, much advancement is set for Alzheimer’s treatment and research. Among the new treatment options, there are targeting butyrylcholinesterase, tau proteins, and endocannabinoid system involvement. The role of microRNAs in AD research. Advanced diagnostic techniques, including qRTPCR and iPSCs, alter early detection (Sharma et al., 2024). The focus has shifted to studies on prodromal phases and biomarkers for early detection of the disease at preclinical levels (Rayathala et al., 2022). The goal of future drugs is the modulation of pathology via alterations to amyloid beta plaques and neurofibrillary tangles, whereas nowadays therapies are mainly trying to level neurotransmitter abnormalities (Ramachandra et al., 2021). To better, the complex causative basis of Alzheimer’s, multiple metabolites like inflammation, the microbiota, hormones, as well as changes in the neurovascular unit, etc., are now being searched for (Giannoni et al., 2020). The discipline is shifting toward personalized medicine, which incorporates different therapies based on individual biomarkers and clinical manifestations (Yiannopoulou and Papageorgiou, 2020). Future Alzheimer’s disease research should prioritize the thorough validation of novel medicines via well-designed clinical trials to ensure reproducibility and effectiveness. Regulatory approvals must include rigorous safety and efficacy studies, particularly for new medicines like psychedelics, stem cells, and nanotechnology-based interventions. Integrating evidence-based conventional treatments is critical for developing holistic approaches that address disease pathophysiology and improve cognitive function. There are still several gaps in understanding the multiple causal metabolites of Alzheimer’s disease, including neuroinflammation, gut-brain interactions, hormone imbalances, and alterations in neurovascular variables. Further research in new diagnostic tools, such as qRT-PCR and iPSCs, for earlier detection of AD, together with the development of biomarkers to allow for personalized treatment regimens, shall be promising in the future. Standardization of protocols, drug delivery systems, and combination therapies will be the keys to further improvements in AD treatment.

### 9.1 Limitations and future research priorities

Despite the promising evidence presented for the use of TCIM in managing Alzheimer’s disease (AD), several limitations hinder its clinical translation. A major concern is the lack of large-scale, multicenter randomized controlled trials (RCTs) that would provide robust evidence on safety and efficacy. Many existing studies suffer from small sample sizes, inadequate control groups, short follow-up durations, and variability in herbal formulations. The bioavailability and pharmacokinetic profiles of key herbal agents, such as curcumin and bacosides, remain suboptimal, restricting their clinical effectiveness.

Standardization of herbal products is also a critical issue. Variability in active compound concentrations due to differences in cultivation, harvesting, and processing conditions can lead to inconsistent therapeutic outcomes. Moreover, herb-drug interactions are not well-characterized, posing potential safety risks when TCIM is combined with conventional therapies.

Future research should prioritize:

• Rigorous, multicenter RCTs with standardized interventions and outcome measures.
• Development of novel drug delivery systems (e.g., nanocarriers) to improve bioavailability.
• Comprehensive studies on pharmacokinetics and herb-drug interactions.
• Identification and validation of biomarkers to assess treatment response.
• Integration of personalized medicine approaches, leveraging genomics and neuroimaging data.

## 10 Conclusion

In conclusion, Alzheimer’s disease is a very complex and fast-growing global health challenge; hence, an innovative and holistic solution is required. TCIM is a way that might be adopted as a strategy for the nature of the disease; current pharmaceutical treatments offer little symptomatic relief for the condition. Key pharmacologically active agents derived from TCIM, such as *C. longa* L. — Family: Zingiberaceae, *B. monnieri* (L.) Wettst. — Family: Plantaginaceae, *Centella asiatica* (L.) Urb. — Family: Apiaceae, and *Withania somnifera*, exhibit multi-targeted mechanisms, ranging from PI3K/Akt and Nrf2 pathway activation to tau aggregation inhibition and cholinergic enhancement. These compounds demonstrate potential for disease modification in Alzheimer’s through antioxidant, anti-inflammatory, and neuroprotective actions. Future integration of such agents into conventional frameworks may open new avenues for holistic yet mechanism-based Alzheimer’s therapeutics.

Collaboration between conventional scientists and TCIM practitioners is pivotal to optimizing TCIM’s strengths in Alzheimer’s treatment. Efficacy, safety, and long-term outcomes will have to be validated by significant, well-planned clinical trials on established protocols. Such an integration will hasten the move of TCIM from adjunct therapies to an established part of mainstream Alzheimer’s care.

Emerging therapeutics include multi-targeted approaches, gene therapy, and nanotechnology. New techniques must be established and tested scientifically and in a multidisciplinary collaboration with other traditional healthcare practitioners before they are included in conventional healthcare. Early diagnosis, specific care, and new treatments might reduce the effects of Alzheimer’s disease in the future and may even help improve people’s lives.
